# Three decades of tuberculosis research in Qatar (1993–2022): A scoping review and future direction

**DOI:** 10.5339/qmj.2026.38

**Published:** 2026-06-21

**Authors:** Nahlah AlMesbah, Reshma Javed, Abid Saeed, Hamad Eid Al-Romaihi, Devendra Bansal, Iheb Bougmiza

**Affiliations:** 1 Preventive Medicine Division, Hamad Medical Corporation, Doha, Qatar; 2 Health Protection and Communicable Disease Control Department, Ministry of Public Health, Doha, Qatar; 3 Preventive and Community Medicine Residency Program, Primary Health Care Corporation, Doha, Qatar; 4 Faculty of Medicine, Sousse University, Sousse Governorate, Tunisia; 5 College of Medicine, QU Health, Qatar University, Doha, Qatar

**Keywords:** Tuberculosis, infectious disease, drug-resistant tuberculosis, migrant health, pediatric tuberculosis, Qatar

## Abstract

**Background:**

Tuberculosis (TB) remained the deadliest infectious disease in 2023, with 8.2 million newly diagnosed cases—the highest number recorded since 1995. In Qatar, the estimated TB incidence was 37 per 100,000 population in 2024. This review aimed to identify gaps in TB research conducted in Qatar and inform future research priorities.

**Methods:**

PubMed, Scopus, Web of Science, and the Cochrane Library were systematically searched using relevant Medical Subject Headings (MeSH) terms and keywords. Peer-reviewed original research articles on TB with data from Qatar published between 1993 and 2022 were included, while reviews and editorials were excluded.

**Results:**

Thirty-six studies met the inclusion criteria. Most were retrospective observational studies focusing on extrapulmonary TB and used nonprobability purposive sampling. The most common research theme was clinical and diagnostic research.

**Conclusion:**

Key research gaps include the evaluation of TB treatment, the epidemiology of drug-resistant TB, and delays in TB diagnosis. The absence of studies on pediatric TB highlights the need to prioritize this population. Strengthening TB research in Qatar will require dedicated funding and a focus on underexplored and high-priority areas.

## 1. INTRODUCTION

Tuberculosis (TB) is a bacterial infection caused by Mycobacterium tuberculosis and other members of the M. tuberculosis complex. It spreads through the inhalation of airborne droplet nuclei generated when a patient with pulmonary or laryngeal TB coughs, sneezes, sings, or shouts.^1^ TB remained the leading infectious disease-related cause of death in 2023. According to the World Health Organization (WHO), 2023 recorded the highest number of newly diagnosed TB cases (8.2 million) since 1995,^2^ of which 8.6% occurred in the Eastern Mediterranean region.^3^ In Qatar, the TB incidence rate was 41 per 100,000 population in 2012, with non-Qataris accounting for approximately 90% of reported cases.^4^ More recent World Bank data indicates a decline to 37 per 100,000 in 2024.^5^ Among Gulf Cooperation Council (GCC) countries, Qatar reported the highest TB incidence in 2024, followed by Bahrain (12 per 100,000), Oman (11 per 100,000), Kuwait (10 per 100,000), the Kingdom of Saudi Arabia (KSA, 8 per 100,000), and the United Arab Emirates (UAE, 1 per 100,000).^5^

An important pillar of the WHO End TB strategy is the prevention of active disease through the treatment of latent TB infection (LTBI), a condition in which M. tuberculosis persists in the host without causing symptoms.^6^ Although LTBI is not contagious, 5–10% of the infected individuals progress to active TB within five years of initial infection.^7^ In Qatar, a study among healthcare workers reported an LTBI prevalence of 3% based on QuantiFERON testing.^8^

According to the United States Centers for Disease Control and Prevention (US-CDC), major TB risk factors include close contact with individuals with active TB, migration from high-burden countries, and residence or employment in hospitals, prisons, shelters, or long-term care facilities.^9^ In Qatar, most TB cases occur among expatriates originating from high TB-burden countries such as India, Bangladesh, and Sri Lanka.^10^ To mitigate these risks, all expatriates applying for residency or visas exceeding one month are required to undergo medical screening, including medical history assessment, physical examination, and chest X-ray, at the Department of Medical Commission, Ministry of Public Health.^11^ Additionally, the Ministry of Interior operates Qatar Visa Centers in selected Asian countries, allowing medical examinations to be completed prior to arrival in Qatar.^12^

Qatar’s National TB Control Program follows WHO guidelines, with management led by the Communicable Disease Center at Hamad Medical Corporation and supported by a state-of-the-art TB laboratory for diagnostics. By law, TB diagnosis and treatment are centralized within the public health sector to ensure adherence to national protocols, and all TB-related healthcare services are provided free of charge.^13^

This scoping review was conducted in alignment with the WHO’s “End TB Strategy,” which emphasizes the importance of research and innovation in achieving global TB elimination targets.^14^ The aim of this review was to map existing TB research conducted in Qatar between 1993 and 2022, identify knowledge gaps, and highlight areas requiring prioritization for future studies.

## 2. METHODS

### 2.1. Search strategy

A comprehensive search was conducted across four electronic databases, including PubMed, Scopus, Web of Science, and the Cochrane Library. The search terms used included “tuberculosis,” “TB,” “tuberculous,” and “Qatar.” These keywords were selected to encompass a wide range of TB-related research articles from Qatar.

### 2.2. Eligibility criteria and study selection

**Figure 1.**
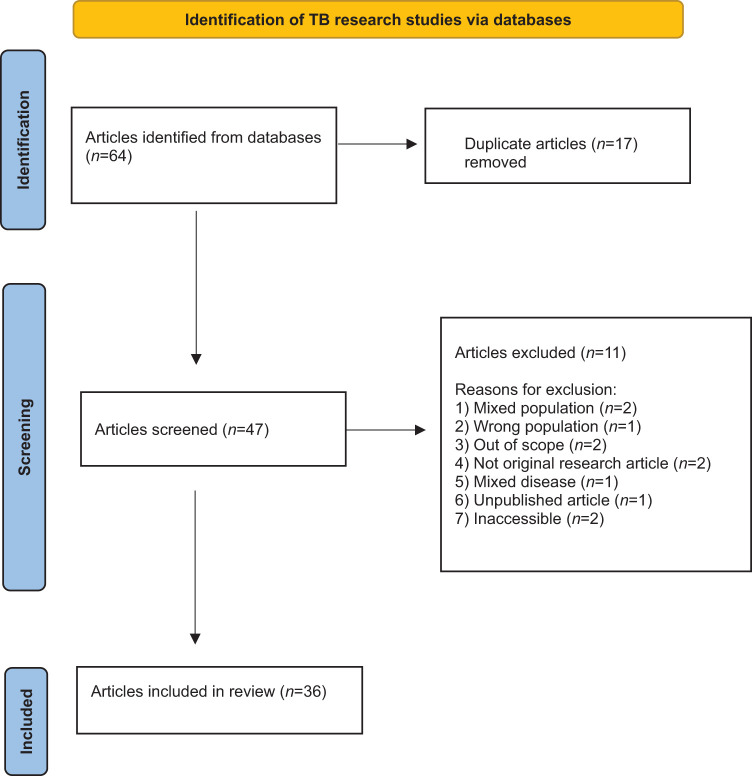
PRISMA diagram. Note: The study with a “wrong population” involved patients diagnosed with TB in Malaysia. The two studies with a “mixed population” included data from GCC countries and were, thus, not specific to Qatar. The two studies classified as “out of scope” were not related to TB. The two “inaccessible” articles were those for which the full text could not be obtained by the reviewers. The study with “mixed disease” referred to an article on communicable diseases that did not specifically focus on TB.

Eligible studies were peer-reviewed original research articles that included data from Qatar and were published between 1993 and 2022. Review articles, editorials, and commentaries were excluded. The article screening and selection process was managed using Rayyan, a web-based tool developed by the Qatar Computing Research Institute to facilitate systematic and scoping reviews. A team of nine reviewers from the Ministry of Public Health, Qatar, independently screened the titles and abstracts. After removing duplicates, 47 of the 64 identified articles were screened for eligibility (Figure 1). Each article was reviewed by at least two team members. Discrepancies were resolved through discussion, and in cases of disagreement, the team leader made the final decision after reviewing the reviewers’ justifications.

### 2.3. Data extraction and analysis

The first author conducted a detailed review of all included studies and extracted relevant data into a structured Microsoft Word table. The extracted variables included author information, study objectives, study design, timeline, population, publication year, sample size, sampling technique, outcomes, limitations, funding status, recommendations, and overarching research themes. Data was summarized using descriptive statistics and presented as frequencies.

## 3. RESULTS

The initial database search identified 64 articles, which were reduced to 47 after removing duplicates. Following screening, 11 articles were excluded for not meeting the inclusion criteria. As a result, a total of 36 studies conducted in Qatar and published between 1998 and 2020 were included in this review (Figure 1).

Of the included articles, 23 were retrospective observational in design, comprising 20 record-based studies,^11,15–33^ two retrospective cohort studies,^34,35^ and one case–control study.^36^ Additionally, there were five prospective observational studies,^37–41^ one cross-sectional study,^42^ six case reports,^43–48^ and one case series.^49^ The sample sizes varied across the studies. For the retrospective studies, the sample size ranged from 8 to 3,301 participants,^11,20^ while prospective studies included 35 to 200 participants.^37,38^ The cross-sectional study included 2,744 participants.^42^ All studies employed non-probability purposive sampling, except for two that used alternative sampling approaches.^36,41^

In terms of study populations, 10 studies focused on pulmonary TB,^11,22,27,32,34–36,40,41,44^ 19 on extrapulmonary TB,^15–18,20,21,23–26,33,38,39,43,45–49^ three included both forms,^29–31^ and one study investigated miliary TB. Two studies were conducted among patients with pleural effusion; however, the results are still pertinent to TB patients.^28,37^ One study was conducted among garment factory workers to determine the prevalence of TB in this population.^42^ Additionally, two of the studies that included both pulmonary and extrapulmonary TB specifically focused on pediatric patients (0–14 years).^30,31^

The extrapulmonary TB studies covered a broad range of manifestations, including pleural TB and tuberculous pleural effusion, spinal TB, abdominal and peritoneal TB, TB adenitis, pancreatic TB, breast TB (mastitis), central nervous system TB (including tuberculoma of the cavernous sinus, primary pituitary TB, and TB meningitis), and TB retropharyngeal abscess.

Thematic analysis revealed a broad distribution of research areas, including epidemiology, antimicrobial resistance, surveillance, health systems, clinical and diagnostic research, and treatment evaluation. Several articles addressed multiple themes (Table 1). The most commonly reported limitations were retrospective study design,^17,24,25,28,29,33–36^ small sample sizes,^24,25,33,35^ lack of follow-up,^34,45,46^ and underreporting of prior TB infection.^11,27^ The number of authors per study ranged from 1 to 13, and study durations varied from 1 to 14 years, with most (eight studies) spanning seven years.^16,17,19,22–24,32,33^ Publications peaked between 2013 and 2020, during which 18 studies were published. Only five studies reported funding support.^11,28,32,39,49^

## 4. DISCUSSION

Although countries in the GCC region have achieved substantial progress toward the WHO End TB targets, including high treatment success rates and comprehensive TB services, variations in incidence trends, mortality, and drug resistance highlight ongoing challenges.^50^ In Qatar, TB is predominantly diagnosed among immigrants from high TB-burden countries moving to Qatar. A study conducted at the Communicable Disease Centre (CDC) in Qatar examined TB patients registered from 2010 to 2015, and found that 97% of the 3,301 culture-positive TB cases were expatriates.^11^ Therefore, identifying research gaps and highlighting priority areas for future research is vital to better understand the disease, guide future interventions, and strengthen national TB control. This scoping review systematically mapped TB research conducted in Qatar between 1993 and 2022, highlighting key thematic trends, research gaps, and opportunities for future investigations.

The studies included in this review covered diverse themes, including clinical and diagnostic research, antimicrobial resistance, epidemiology, and health systems research. These areas reflect ongoing challenges in TB detection, diagnosis, and management, particularly in the context of antimicrobial resistance, where continuous surveillance remains vital.

A global survey of TB-related systematic reviews by Nicolau et al.^51^ found that “detection, screening, and diagnosis” was the most common research theme (32.6%), consistent with our findings, where 44% of TB studies in Qatar focused on diagnostics. Similarly, Benabdellah et al.^52^ identified this as the second most common theme (25%). However, while international studies report a higher emphasis on treatment evaluation—ranging from 23.4% to 30%.^51,52^ Only 5.6% of the studies conducted in Qatar addressed treatment outcomes. This represents a key research gap, as evaluating therapeutic regimens has significant clinical and financial implications for both national and global TB control programs.

Our results also align with previous reviews regarding “epidemiology” as a recurring theme, corresponding to the “TB etiology and risk factors” identified by Nicolau et al.^51^ In contrast, none of the studies conducted in Qatar addressed TB prevention, differing from the findings of Benabdellah et al.^52^ This gap likely reflects the unique epidemiological context of Qatar, where most TB cases occur among expatriates. Preventive strategies in this setting largely depend on mandatory pre-arrival or entry screening, which is strictly implemented. However, this approach has inherent limitations, as expatriates may repeatedly travel to high TB-burden countries after obtaining residency. Thus, new infections may occur long after the initial screening, limiting the effectiveness of one-time, entry-based preventive strategies.

Drug-resistant TB (DR-TB) research remains limited in Qatar, with only two relevant studies identified (2001 and 2020).^11,27^ In comparison, a systematic review from KSA reported 22 DR-TB articles published between 1979 and 2013,^53^ with an overall MDR-TB (multidrug resistant tuberculosis) prevalence of 6.7% compared to 1.2% in the 2020 Qatar study.^11,27,53^ Given Qatar’s centralized TB management at the Communicable Disease Center, periodic national-level studies are feasible and should be conducted more regularly, ideally every few years, to facilitate the dissemination of DR-TB trends in the scientific literature.

We identified one study on delays in TB diagnosis and management conducted in Egypt by Elsaid et al.^54^ and two studies conducted in KSA by Almohaya et al.^55^ and Dahmash et al.^56^ Compared to the single study from Qatar, KSA’s studies are from various regions of the country, reflecting its larger geographic area. One study from KSA by Almohaya et al.^55^ examined diagnostic delays among inpatients and found an average delay of 5.5 days. The study identified in our review found average delays of 45.7 days and 46.3 days due to patient-related and health system-related factors, respectively.^41^ Additionally, the Egyptian study by Elsaid et al.^54^ focused on the delay in TB management, which may be unnecessary in Qatar, given that there are no delays and the treatment is provided free of charge. It should be noted, however, that the study conducted in Qatar was published in 2016; thus, an updated assessment, especially delays related to health system factors, would be useful.

Pediatric TB research is another area of deficiency. Only two pediatric studies were identified: one investigating erythrocyte sedimentation rate as a diagnostic marker and another describing TB epidemiology between 1983 and 1996.^30,31^ Both studies are outdated and insufficient to reflect the current pediatric TB situation. Given that pediatric cases are managed at both Sidra Medicine and the Communicable Disease Center, future studies should focus on this population to better understand disease burden, clinical presentation, and outcomes.

## 5. STRENGTHS AND LIMITATIONS.

This review represents the first comprehensive synthesis of all TB research conducted in Qatar. Data extraction by a single reviewer ensured consistency across all included studies. However, potential biases may have been introduced during data abstraction, particularly as some dates lacked specific months, leading to assumptions of study duration. Additionally, the review relied solely on the limitations explicitly reported in the included studies, which may have excluded unacknowledged methodological constraints.

## 6. CONCLUSION AND WAY FORWARD

This scoping review provides a comprehensive synthesis of TB research conducted in Qatar between 1993 and 2022. Although Qatar has demonstrated a gradual decline in TB incidence from 41 per 100,000 population in 2012 to an estimated 37 per 100,000 in 2024, it continues to report a high TB incidence among GCC countries, with the vast majority of cases diagnosed among expatriates traveling to Qatar from high-burden regions.^4,5^ In this context, understanding the scope and focus of national TB research is essential to inform future control efforts.

TB research in Qatar has been largely retrospective and focused on diagnostic and epidemiological themes. While these studies have contributed valuable insights into disease patterns and clinical manifestations, significant gaps remain. Treatment evaluation studies are limited, DR-TB research has been infrequent, and only one study has assessed diagnostic delay. Pediatric TB is markedly underrepresented, with only two outdated studies identified. In addition, no studies have addressed TB prevention strategies, reflecting an area requiring further investigation given Qatar’s reliance on entry-based screening in a highly mobile expatriate population.

Addressing these gaps through prospective research, periodic drug-resistance surveillance, updated pediatric studies, and evaluations of treatment outcomes and diagnostic pathways will be important to strengthen evidence-informed policy. Leveraging Qatar’s centralized TB care system and expanding structured research support will be essential to sustain progress and advance toward the WHO End TB Strategy targets.

## ACKNOWLEDGEMENT

We acknowledge the valuable contribution of the Health Protection & Communicable Disease Control team to the article screening process.

## AUTHORS’ CONTRIBUTION

NA: Conceptualization, review of all included studies, data curation, analysis, writing of the original draft, and editing. RJ, AS, HA, and DB: Review and editing. IB: Supervision, review, and editing.

## DISCLOSURE OF AI USE

ChatGPT was used for language enhancement only.

## CONFLICT OF INTEREST STATEMENT

The author(s) declare that there is no conflict of interest.

## Figures and Tables

**Table 1.** Outcomes/objectives, sample size, and timeline categorized by study themes.

Authors	Outcomes/objectives	Sample size	Timeline
**Epidemiology/antimicrobial resistance/surveillance**			
Al-Marri^27^	Determined the resistance patterns of Mycobacterium tuberculosis to four anti-TB drugs in pulmonary TB following the implementation of DOTS and found that 61 cases (15%) were resistant to one or more anti-tuberculous drugs.	406	January 1996 to December 1998
Ali^11^	Described the epidemiology of drug-resistant TB in Qatar and reported that 223 cases (6.7%) exhibited resistance to at least one drug, while 38 cases (1.2%) exhibited resistance to multiple drugs. The study also mentioned that being a former resident of India is a risk factor and that cure rate was 97.6% and relapse rate was 2.4%.	3,301	January 2010 to March 2015
**Epidemiology/health systems research**			
Ibrahim^41^	Diagnostic delay among pulmonary TB patients was studied and it was found that health system factors were associated with more cases of delay (45%) compared to patient factors (35%). The mean health system factor delay was 46.3 days, and the median was 30 days. The mean patient factor delay was 45.7 days and the median was 30 days.	100	January 2007 to December 2015
**Epidemiology/clinical research**			
Imam^24^	Described the clinical presentation, diagnosis, treatment, and outcomes of patients with tuberculous meningitis. The author also concluded that the incidence of adult tuberculous meningitis was 0.9 per 100,000 population between 2006 and 2012.	80	January 2006 to December 2012
Al Soub^26^	Discussed the clinical features and factors affecting outcomes in TB meningitis.	20	January 1990 to December 1995
**Epidemiology**			
Ibrahim^23^	Determined that 85% of individuals affected by TB were below the age of 46 years. The author also concluded that pleural TB tended to be a primary disease rather than a reactivation of parenchymal disease.	100	January 2007 to December 2015
Al-Marri^31^	Described the decreasing incidence of TB among the pediatric population between 1983 and 1996, from 11 to 7 cases per 100,000 children.	144	1983 to 1996
Zahid^15^	Described the profile of patients with tuberculous pleural effusion in Qatar and stated that the condition was more common among young, healthy adults from countries with a high TB burden. The authors also concluded that low BMI was not a risk factor for tuberculous pleural effusion.	100	January 2016 to December 2019
Al-Khal^42^	Studied the prevalence of TB among garment factory workers and found it to be 43%.	2,774	January 2000 to June 2003
Abu Khattab^29^	Described the demographic characteristics of TB patients in Qatar.	1,221	January 2005 to December 2008
Khan^37^	Found that tuberculosis was the most common cause of pleural effusion in Qatar (32.5%).	200	January 2005 to December 2005
**Clinical research/clinical course**			
Al Soub^43^	Described the clinical course of a case of tuberculoma of the cavernous sinus, from diagnosis through treatment to complete resolution.	1	N/A
Thomas^44^	Described the clinical course of a 58-year-old man who presented with tuberculosis and febrile neutropenia.	1	N/A
Ali^45^	Described a rare case of pancreatic TB which was diagnosed based on histology and microbiology, even though the QuantiFERON was negative.	1	N/A
Ben Abid^46^	Described the clinical course of a case of primary pituitary TB that was diagnosed histologically.	1	N/A
Ben Abid^47^	Described a case of culture-negative TB mastitis that may be easily confused with idiopathic granulomatous mastitis, and highlighted the importance of differentiating between the two, as idiopathic granulomatous mastitis is treated with steroids, which are contraindicated in TB as they may cause the condition to worsen.	1	N/A
Goravey^49^	Described three cases of extrapulmonary tuberculosis presenting as a chest wall abscess, appendicitis, and a ganglion.	3	N/A
Radi^48^	Described a case of tuberculous retropharyngeal abscess.	1	N/A
**Clinical research/treatment evaluation**			
Al Shaer^35^	Compared fixed-dose combinations with separate-tablet regimens in patients with pulmonary TB. Showed that there is no difference in the outcomes between the two regimens among otherwise healthy TB patients. Also showed faster sputum conversion among diabetic patients who received fixed-dose combinations compared to those on separate-tablet regimens.	148	December 2012 to November 2014
Al Shaer^34^	Compared fixed-dose combinations to the separate-tablet regimens in patients with pulmonary TB. Showed faster sputum conversion among diabetic patients who received fixed-dose combinations compared to those on separate-tablet regimens.	103	December 2012 to December 2015
**Clinical research/diagnostic research**			
Al-Marri^30^	Conducted on pediatric patients and concluded that erythrocyte sedimentation rate was not a useful diagnostic test.	144	1983 to 1996
Al Alousi^32^	Assessed whether a third sputum sample was necessary for the diagnosis of active pulmonary TB and concluded that two samples are adequate.	687	January 2002 to December 2008
Alhowady^40^	Tested the hypothesis that TB patients are not infectious two weeks after starting anti-TB medications and found that 95.7% of the participants tested positive on sputum culture after two weeks of anti-TB treatment initiation; thus, concluded that it is unsafe to discontinue isolation.	95	November 2013 to November 2014
Imam^33^	Evaluated the accuracy of the Thwaites Diagnostic Score and the Lancet Consensus Score in the diagnosis of TB meningitis and found that a Lancet Consensus Score >12 was diagnostic.	156	2007 to 2014
Khan^39^	Investigated whether interferon-gamma and adenosine deaminase could be used to differentiate between tuberculous and nontuberculous pleural effusions and concluded that interferon-gamma is more sensitive and specific for tuberculous effusions than adenosine deaminase.	103	June 2009 to May 2010
Thomas^28^	Determined that the diagnostic yield of medical thoracoscopy for exudative pleural effusion was 91.4%; however, the study recommended that closed needle biopsy be performed initially for financial and safety purposes.	407	January 2008 to December 2015
Howady^16^	Described experience with spinal tuberculosis in Qatar and concluded that chronic back pain should be investigated using CT/MRI, followed by CT-guided fine-needle aspiration.	35	1992 to 1998
Szmigielski^18^	Recommended the use of CT and ultrasound during the workup of abdominal TB, but acknowledged that microbiology/histology is ultimately needed for diagnosis. Further added that a contrast study is needed to assess the extent of the disease for surgical purposes.	59	1987 to 1995
Abdelaal^17^	Described experience with peritoneal TB and concluded that laparoscopic peritoneal biopsy is fast and accurate compared to a microbiological diagnosis, which may take 4–6 weeks.	41	January 2004 to December 2010
Khan^25^	Concluded that laparoscopic peritoneal biopsy is fast and accurate compared to a microbiological diagnosis for peritoneal TB, and added that six months of treatment with a four-drug regimen improved outcomes.	54	2005 to 2009
Al Soub^19^	Concluded that a biopsy is often needed to make a histological diagnosis in miliary TB to allow commencement of treatment, as AFB smears are commonly negative and microbiological diagnosis is often delayed.	32	1992 to 1998
Khan^38^	In a study among TB adenitis patients, it was found that lymph node excision and fine-needle aspiration had similar microbiological yields, but lymph node excision had a higher histological yield.	35	January 2006 to December 2006
Al-Marri^21^	Concluded that a biopsy is required to make a histological diagnosis of breast TB, as clinical and radiological features are not specific.	13	1988 to 1998
Al-Marri^20^	Mammographic features of TB mastitis were reviewed, and it was concluded that mammography is not helpful in differentiating TB from breast carcinoma, and that histological diagnosis remains the gold standard.	8	1990 to 2002
Dousa^36^	Compared diabetics with TB to nondiabetics with TB and found that 53% of the diabetic patients continued to have positive sputum cultures two months after anti-TB treatment initiation, compared to 27% of patients without diabetes.	268	January 2007 to December 2011
Al-Marri^22^	Evaluated the tuberculin skin test and concluded that clinical judgment should always be applied, as 9.8% of confirmed pulmonary TB cases had false-negative results.	306	1998 to 2004

DOTS, Directly observed treatment short course; BMI, Body mass index; CT, Computed tomography; MRI, Magnetic resonance imaging; AFB, Acid-fast bacillus.
